# Purple potential in petunias: flavonoid pathway related regulator evolution

**DOI:** 10.1093/plphys/kiag180

**Published:** 2026-04-07

**Authors:** Lisa Oskam

**Affiliations:** Assistant Features Editor, Plant Physiology, American Society of Plant Biologists, Rockville, Maryland 20855-2768, United States; Laboratory of Molecular Biology, Wageningen University, Wageningen, the Netherlands

Plants synthesize a wealth of different pigments, coloring our everyday surroundings through flowers, fruits, and vegetables. These pigments include flavonols and other derivatives of the flavonoid biosynthesis pathway, such as aurones, anthocyanins, and tannins (Albert et al. 2022). The precursors stem from phenylalanine through the phenylpropanoid pathway, culminating in the biosynthesis of chalone, of which flavonoids are derived. Betalains and carotenoids are the 2 other major classes of pigments and derive from tyrosine and isopentenyl pyrophosphate, respectively (Tanaka et al. 2008). Plants utilize these pigments not only to attract pollinators and seed dispersers but also as photoprotection to aid in resistance against abiotic and biotic stresses and even in rhizobia symbiosis (Albert et al. 2022).

Generally, it is thought that the evolution of flavonoid biosynthesis coincided with the terrestrialization of plants. Presumably, exposure to the new surrounding biotic and abiotic stresses drove the expansion of both the pigments and the genetic regulation through the evolutionary branches (de Vries and Feussner 2024). Various branches of the flavonoid biosynthesis pathway are regulated through a host of different transcription factors and co-regulators that are tissue- and timing-context dependent. Members of the R2R3-MYB-domain transcription factors are involved in the transcriptional activation and repression of key enzymes in branches of the flavonoid pathway and are often the common denominator (Zhu et al. 2025). R2R3-MYBs often function in larger complexes, together with basic-Helix-Loop-Helix (bHLH) and WD40 proteins (MBW). With terrestrialization came diversification and the development of new land plant lineages, which coincides with the vast expansion of the R2R3-MYBs in various subgroups (SGs) (Wu et al. 2022). Whole genome and gene duplications enabled broadening of the R2R3-MYB palette, not only in number but also in terms of downstream regulation and protein interactions. In a recent *Plant Physiology* paper, Zhang and colleagues (2026) described a series of experiments to determine the cause of the functional differences between MYBs that regulate distinct branches of the flavonoid pathway in an evolutionary context.

Phylogeny analyses across a variety of lineages of pigment-related R2R3-MYBs by Zhang and colleagues (2026) resulted in SGs that are separated on basis of functionality related to specific branches of flavonoid biosynthesis. Zhang and colleagues employed the model system *Petunia axillaris* × *Petunia hybrida* F1 to test the potential of different MYBs from different backgrounds to induce anthocyanin biosynthesis. This cross carries null mutations in the 2 MYBs that are in SG6, which are involved in the regulation of anthocyanin biosynthesis (Fig. 1), rendering the petunia petals white. Reactivation of anthocyanin accumulation results in a purple phenotype. The authors found that both transient expression and expression through stable transformants of representatives of the different SGs activated partially overlapping and distinct gene sets (Fig. 1). MYBs belonging to the same SG but stemming from a different species displayed a substantial overlap with their petunia counterpart. Additionally, SG4 and SG6 both repressed the expression of genes specific to the anthocyanin or the flavonol and tannin pathway, respectively (Fig. 1).

**Figure 1:**
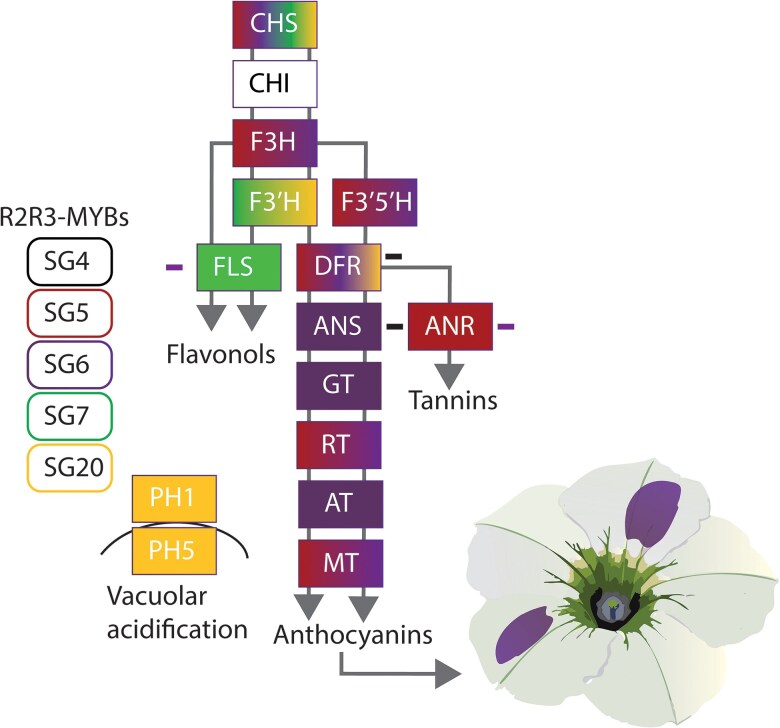
R2R3-MYBs expression regulation of flavonoid biosynthesis genes in *Petunia hybrida*. Five subgroups (SGs) as defined by Zhang and colleagues (2026) were represented by either a single representative R2R3-MYB from petunia or a single representative from another species. Colors of gene boxes represent strong induction of gene expression after either transient expression or stable transformation of a color corresponding SG R2R3-MYB in the study of Zhang and colleagues (2026). Mild induction is not represented here. Minuses next to genes indicate a downregulation after either transient expression or stable transformation of a color corresponding SG R2R3-MYB. Purple spots on petunia petals represent successful anthocyanin accumulation. Gene abbreviations: CHS, Chalcone Synthase; CHI, Chalcone Isomerase; F3H, Flavanone 3-Hydroxylase; F3′H, Flavonoid 3′-Hydroxylase; F3′5′H, Flavonoid 3′,5′-Hydroxylase; FLS, Flavonol Synthase; DFR, Dihydroflavonol 4-Reductase; ANR, Anthocyanidin Reductase; ANS, Anthocyanidin Synthase; GT, Flavonoid Glycosyltransferase; RT, Flavonoid 3-O-Glucoside-Rhamnosyltransferase; AT, Acyltransferase; MT, Methyltransferase; PH1, PH5, P-ATPASE 1.

Petunia petals express several MYB partners that can form the MBW complexes with MYBs; however, petunia leaves lack the expression of several MYB partners. The authors utilized the difference in interaction partner expression to test whether SGs differed in their ability to activate the flavonoid biosynthesis pathway. Zhang and colleagues found that indeed SG6 and SG7 were still able to activate genes related to branches culminating in anthocyanins and flavonols, respectively. However, representatives of SG5 and SG20 could no longer induce expression of genes in the flavonoid biosynthesis pathway. MYBs from the same SG but from different species differed in their biding affinity for interaction partners, suggesting divergent evolution for these traits. Together, these results indicate an affinity of different MYB SGs for distinct branches of the flavonoid pathway and potentially redirect biosynthesis toward a specific branch through downregulation of competing branches. Though it is unclear from these experiments whether other interaction partners of MBW complexes play a role in this proposed antagonistic gene regulation.

Lastly, Zhang and colleagues (2026) studied the sequence of SG6 MYB ANTHOCYANIN SYNTHESIS REGULATOR 1 (ASR1) in more detail. Chimeras of N and C terminus from SG6 ASR1 and other MYB SGs indicated that despite highly divergent C-terminal sequences between a SG6 and a SG20 MYB, the activation level of gene expression remained the same. Amino acid substitutions in the N-terminal region of ASR1 resulted in the loss of anthocyanin accumulation, highlighting the importance of the evolutionary conserved region for successful flavonoid biosynthesis.

This study by Zhang et al. (2026) cleverly exploits the petunia model system to study functional divergence in a large gene family. Further research into the motifs of R2R3-MYBs involving both the interactions with MBW partner proteins and cis-regulatory elements could provide a deeper understanding of the functional evolution of these MYBs in different lineages. Better understanding of sequence diversity in terms of functional implications would also aid in the continuing development of metabolic engineering.

Further reading in *Plant Physiology*:

1. Yuan et al. (2025) studied flavonoid biosynthesis-related R2R3-MYBs structures and their functional implications in strawberry.
2. Zhu et al. (2025) provided an overview of the recent advances in biosynthesis, and molecular regulation of flavonoids, combined with an emphasis on metabolic engineering.

## Data Availability

The data underlying Fig. 1 in this article are available in Zhang et al., 2026.
